# CAN SOCIAL ENVIRONMENT OFFSET THE GENETIC RISK OF MYOCARDIAL INFARCTION AMONG OLDER ADULTS?

**DOI:** 10.1093/geroni/igac059.1923

**Published:** 2022-12-20

**Authors:** Chenkai Wu, Chen Sheng

**Affiliations:** Duke Kunshan University, Kunshan, Jiangsu, China (People's Republic); Fudan University, Shanghai, Shanghai, China (People's Republic)

## Abstract

The interrelatedness between social determinants of health impedes researchers to identify important social factors for cardiovascular health. Additionally, it remains largely unknown whether a derivable social environment could offset the genetic risk for cardiovascular events. We developed a polysocial score approach to quantify the aggregate effect of social factors on myocardial infarction (MI). We also examined the association of polysocial score and polygenic risk scores (PGS), and their interaction, on MI. Data are from the Health and Retirement Study, a longitudinal cohort of a nationally representative sample of Black and White Americans with pre-calculated PGS for MI (N=6,036). We included 24 social factors from five categories (economic stability, neighborhood environment, education, community, and social context, and healthcare system) and used forward stepwise regression to screen for important ones. Polysocial score was created using 14 social factors and was classified as low (< 28), intermediate (29-39), and high (40+). The incidence of MI was 4.5, 8.5, 10.5 per 1000 person-years among Whites with a low, intermediate, and high PGS, respectively; no graded association was found among Blacks. Polysocial score stratified the rate of MI in each tertile of PGS among Whites. We found a significant additive interaction between PGS and polysocial score. The difference in MI rate was 10.3 per 1000 person-years among individuals with a high genetic risk, while the difference significantly reduced to 3.5 per 1000 person-years among those with a low genetic risk. Desirable social environment could possibly offset the increased risk of MI associated with genetics among Whites.

